# Lead and cadmium biosorption from milk by *Lactobacillus acidophilus* ATCC 4356

**DOI:** 10.1002/fsn3.1825

**Published:** 2020-08-18

**Authors:** Ramona Massoud, Kianoush Khosravi‐Darani, Anoosheh Sharifan, GholamHassan Asadi, Allaleh Zoghi

**Affiliations:** ^1^ Department of Food Science Standard Organization Tehran Iran; ^2^ Research Department of Food Technology Faculty of Nutrition Sciences and Food Technology National Nutrition and Food Technology Research Institute Shahid Beheshti University of Medical Sciences Tehran Iran; ^3^ Department of Food Science and Technology, Science and Research Branch Islamic Azad University Tehran Iran

**Keywords:** bioremoval, cadmium, *Lactobacillus acidophilus*, lead, milk

## Abstract

The food and water contamination with heavy metals is increasing due to the environmental pollutions. Lead and cadmium are the toxic heavy metals for humans that can be found in air, soil, water, and even food. Lactic acid bacteria have the ability to remove and diminish the level of heavy metals. In this study, *Lactobacillus acidophilus* was used to remove lead and cadmium in milk and the capability of this valuable bacterium in biosorption of these metals low concentrations (µg/L or ppb) in milk was evaluated. First, the variables on lead and cadmium removal by this bacterium have been studied by Plackett–Burman design. Then, the bioremoval process was optimized and the three main factors, the bacterium concentration, contact time, and the initial heavy metal concentration were chosen by using a central composite design. The optimum lead and cadmium bioremoval yield of 80% and 75% were observed, respectively, at 1 × 10^12^ CFU of *L. acidophilus* in milk at the 4th day and the initial ion concentration of 100 µg/L. The 3D plots analysis showed the interaction effects on metal biosorption. This study showed that *L. acidophilus* is a natural effective biosorbent for lead and cadmium removal from milk.

## Graphical abstract

Lactic acid bacteria have the ability to remove and diminish the level of heavy metals. Biosorption depends on some process variables. *Lactobacillus acidophilus* is a natural effective biosorbent for lead and cadmium removal from milk.

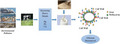

## 1 INTRODUCTION

The food and water contamination with heavy metals is increasing due to the environmental pollutions. Heavy metals are the elements with the density of more than 5 g/cm^3^ (Rajaganapathy, Xavier, Sreekumar, & Mandal, 2011). Heavy metals have become a serious problem as a result of the urbanization and industrialization. These toxic metals pollute water, soil, plants, and eventually foodstuffs and our bodies. Pb and Cd are the toxic heavy metals for humans that can be found in air, soil, water, and even food. They are in the risky heavy metals’ group (Bhakta, Ohnishi, Munekage, Iwasaki, & Wei, 2012; Jin et al., 2019). They are undegradable compounds, and their toxicity in the environment is known as the most dangerous pollutants in the world (Demirbas, 2008). The chemical fertilizers, industrial wastewater, mines are the main sources of releasing Pb and Cd to the environment (Sardar, Hameed, & Afzal, 2013).

Milk as the most common dairy product may be contaminated by various pollutants such as heavy metals, so it is one of the most serious problems for the public health in the world (Derakhshesh & Rahimi, 2012; Fischer, Schilter, Tritscher, & Stadler, 2015). As toxic metals’ presence in milk is so important, thus their levels should be controlled continuously (Semaghiul, Simona, Gabriela, & Alina, 2008).

According to “Codex Standard for Contaminants and Toxins in Food,” the permissible limits for Pb and Cd concentrations in milk should be <20 and 10 µg/L, respectively. Some studies have reported Pb and Cd contamination, respectively, more than these standard levels in milk around the world such as China 40 and 20 µg/L (Qin, Wang, Li, Tong, & Tong, 2009); Iraq 35 and 12 µg/L (Alani & Al‐Azzawi, 2016); Turkey 22 and 14 µg/L (Ayar, Sert, & Akın, 2015); and some cities in Iran such as Isfahan 42 and 16 µg/L; Mashhad 33 and 12 µg/L; Ahvaz 38 and 22 µg/L (Rahimi, 2013).

There are some physiochemical ways for heavy metal detoxification such as ion exchange, chemical precipitation, and membrane technologies but they are not so useful for foodstuffs because they are very costly and not usable in foodstuffs (Kobielsk, Howarth, Farha, & Nayak, 2018; Zabochnicka‐Åšwi & Krzywonos, 2014; Zhao et al., 2018).

The biological methods include applying microorganisms such as bacteria, yeasts, fungi, and algae. These biosorbents are cheap and useful for foods (Wang et al., 2015, 2018) and also suitable for heavy metals’ bioremoval even in very low levels (Massoud, Hadiani, & Khosravi‐Darani, 2019; Massoud, Khosravi‐Darani, Sharifan, & Asadi, 2019). This mechanism reduces the toxic metals through physical adsorption, electrostatic interaction, chelation, and ion exchange in the microorganism's membrane (Wang & Chen, 2009).

Lactic acid bacteria have the ability to remove and diminish the level of heavy metals from aqueous solution (Halttunen, Finell, & Salminen, 2007; Zhai et al., 2015). They are in the list of GRAS (Chen & Narbad, 2018) and also able to bind heavy metals in low concentrations. The negative surface charge of the LABs helps them in binding to metal cations (Zoghi, Khosravi‐Darani, & Sohrabvandi, 2014). Potential of various LABs for the heavy metals bioremoval have been reported such as Pb, Cd removal by *L. bulgaricus* (Chang, Choi, & Kikuchi, 2012), and Cd by co‐culture of *L. plantarum* and *B. coagulans* (Majlesi, Shekarforoush, Ghaisari, Nazifi, & Sajedianfard, 2017), as well as As removal by *L. acidophilus* (Singh & Sarma, 2010), Cd by *L. plantarum* (Hao, Reiske, & Wilson, 2000). Biosorption of Cd and Pb by lactic acid bacteria (Li et al., 2020) and Pb and Cd removal by lactic acid (Halttunen, Salminen, Jussi, Raija, & Kalle, 2008). The gap of research in all these reports seems to be the lack of considering heavy metals’ removal in the range of very small amounts (ppb) in foodstuff and water.

In the previous projects of this experimental team, we used *Saccharomyces cerevisiae* for biosorption of heavy metals from drinking water (Hadiani, Khosravi‐Darani, Rahimifard, & Younesi, 2018) and milk (Massoud, Hadiani, et al., 2019; Massoud, Khosravi‐Darani, et al., 2019; Massoud, Khosravi‐Darani, Sharifan, & Asadi, 2020) and also the biosorption of mercury in milk by the help of *L. acidophilus* (Massoud et al., 2020).

In this study, *Lactobacillus acidophilus* ATCC 4356 was used to remove Pb and Cd in milk due to its popularity in dairy industry, the previous studies and the experiences of our research team, and the capability of this valuable bacterium in biosorption of these metals low concentrations (µg/L or ppb) in milk was evaluated. So, the 5 process variables (contact time, metal concentration, bacterial biomass, inoculation temperature, and shaking rate) were chosen for the Plackett–Burman design (PBD) and distinguishing of main factors. After that, the interaction of main variables (metal concentration, contact time, and biomass concentration) has been studied in 5 levels by response surface methodology (RSM) to reveal the optimum condition of bioremoval. Eventually, the biosorption isotherms (Langmuir and Freundlich models) were applied to clarify the ability of *L. acidophilus* (10^12^ CFU/ml) for Pb and Cd bioremoval at different metal concentrations. Up to now, there is no published report about the biosorption of Pb and Cd by *L. acidophilus* in milk, our project would be the first step in using this profitable bacterium as the biosorbent in foodstuffs.

## 2 MATERIALS AND METHODS

### 2.1 Bacterial strain and chemicals

*Lactobacillus acidophilus* ATCC 4356, the commonly used LAB in dairy industry, was selected and purchased from Tak Gene Zist Company. The bacteria were inoculated in 10 ml of MRS broth and then incubated for 48 hr at 37°C. The bacteria's viability was determined by total plate counting and MRS agar and plate count agar for *L. acidophilus* counting (Vinderola & Reinheimer, 2000).

The standard solution of Pb and Cd (1,000 mg/L, Merck), MRS agar, MRS broth, and plate count agar were prepared from Liofilchem. The other reagents like H_2_O_2_ (Prolabo, Spain), nitric acid (Merck), bovine serum albumin (Labclinic, Spain), and phosphate‐buffered saline (HyClone, Spain) were also used in this work.

### 2.2 Pb and Cd detection by ICP‐MS

The inductively coupled plasma mass spectrometer (ICP‐MS) (England) with a cross flow rate nebulizer and a Peltier‐cooled quartz spray chamber was used in this study. The instrument was checked by the aqueous multielement before each experiment. First, the prepared samples were digested by the microwave with a MPR‐600 rotor (at 35 bar and 260°C) (Khan et al., 2014; Li et al., 2018).

### 2.3 Process variables

All possible process factors which may have an impact on the biosorption yield of Pb and Cd biosorption by *L. acidophilus* were selected based on literature reviews (Halttunen et al., 2008; Jadán‐Piedra et al., 2017; Pakdel, Soleimanian‐Zad, & Akbari‐Alavijeh, 2018; Singh & Sarma, 2010) including the contact time, size of inoculation, metal concentration, temperature and shaking rate. Then, according to our pre‐experiences, the process variables’ interaction effects should be examined. The experimental planning by the help of a screening design was applied, and the significant factors are highlighted. Finally, the central composite design was used for the optimization design of the experiments (Coruh, Elevli, & Geyikçi, 2012).

### 2.4 Plackett–Burman design (PBD)

According to the literature reviews and pre‐experienced trials, the 5 independent variables in Pb and Cd biosorption by *L. acidophilus* were as follows: the contact time, Pb and Cd concentrations, shaking rate, biomass dosage, and inoculation temperature. Table 1 shows PBD for evaluation of these process variables in two experimental levels.

**Table 1:** Plackett–Burman for investigation of the variables’ effect on Metals bioremoval by *Lactobacillus acidophilus*

Run	Bacterial biomass (CFU)	Contact time (day)	Mercury concentration (µg/L)	Inoculation temperature (°C)	Shaking rate (rpm)
1	1 × 10^11^	1	40	4	50
2	1 × 10^12^	4	40	40	0
3	1 × 10^11^	1	40	40	0
4	1 × 10^12^	4	40	4	50
5	1 × 10^11^	4	100	40	50
6	1 × 10^12^	1	100	40	50
7	1 × 10^11^	4	100	4	0
8	1 × 10^12^	1	100	4	0

The aim of our study is to investigate the capability of *L. acidophilus* to eliminate the small amounts of Pb and Cd in milk. The heavy metal concentrations were chosen in very low levels as our teams’ previous study on the drinking water. Then, the project was planned by Design Expert software to verify the maximum removal conditions.

For the designed experiment of Pb and Cd bioremoval in this study, the bacterial biomass (1 × 10^11^ and 1 × 10^12^ CFU) was added to sterile milk at 37°C; then, the samples were rested for 20 min and after that the metals (40 and 100 µg/L) were added and put at on the shaker. In the 1st and 4th day (as the defined contact time), the bacteria cells were centrifuged (at 8,000 × *g* for 20 min). Finally, the supernatants were analyzed for residual Pb and Cd concentrations by using ICP‐MS. All the experiments were done in triplicates. The ability of *L. acidophilus* to absorb Pb and Cd was evaluated as follows:

(1) $$\%\text{Removal}=100\times \left[\left(C_{0}‐C_{1}\right)/C_{0}\right]$$

where *C*_0_ is the initial and *C*_1_ is residual mercury concentration (Zhai et al., 2015).

The collected data were analyzed by Minitab statistical software (version 14). According to the results, 3 main variables were as follows: *L. acidophilus* concentration, the contact time, and metal concentration. For the experimental optimization of this study, the fractional factorial design was performed.

### 2.5 Response surface methodology (RSM)

Response surface methodology is a mathematical technique that helps to analyze the effects of process variables on the responses (Aslan & Cebeci, 2007). The Plackett–Burman results showed that three process variables: metal concentrations, contact time, and *L. acidophilus* concentration had significant effects on Pb and Cd bioremoval. RSM was designed for optimizing the variable levels for Pb and Cd bioremoval and also the minimizing the number of tests. In this project, CCD was applied to investigate the optimum biosorption conditions by the help of the experimental factors shown in Table 2.

**Table 2:** Main process variables and levels for Pb and Cd bioremoval by *Lactobacillus acidophilus by* CCD

Range and level					
Independent variable	− α (−1.68)	−1	0	+1	+ α (+1.68)
*L. acidophilus* concentration (CFU)	1 × 10^10^	10 × 10^11^	1 × 10^12^	10 × 10^13^	10 × 10^14^
Contact time (day)	0	1	2	3	4
Initial metal concentration (µg/L)	40	50	70	90	100

The Design Expert 7.1.5 (Stat‐Ease Inc.) software was applied for designing the run tests and also data analysis. The runs were designed in the 5 levels (Table 2) while the other factors were kept constant as the following: the shaking rate at 50 rpm and the inoculation temperature at 25°C.

### 2.6 Determination of the binding metal capacity

The isotherm models like Langmuir and Freundlich would assist in predicting the maximum ability of binding heavy metals. These absorption models were applied to explain capability of the bacterial cells to absorb Cd and Pb at a predetermined time (Andreasen et al., 2018; Freundlich, 1906; Langmuir, 1918).

### 2.7 Statistical analysis

The statistical analysis was analyzed by MINITAB statistical software (version 14), and then, the response surface plots were designed. The statistical data were prepared by analysis of variance. All data are calculated as the mean value ± standard deviation (*M* ± *SD*) of independent variables in defined days. The *p*‐values < .05 were statistically significant.

## 3 RESULTS AND DISCUSSION

### 3.1 Optimization of Pb and Cd bioremoval by RSM

The analysis of variance represented the effects of the process variables that designed by Plackett–Burman design. Applying RSM after the variance analysis showed that the Pb and Cd biosorption levels are the result of the 3 main variables as shown in Table 3. The *p*‐values <.05 showed that the model terms are significant. In this study, Pb and Cd concentrations, contact time, and biomass dosage are significant model terms.

**Table 3:** Analysis of variance of parameters for Pb (a) and Cd (b) bioremoval by *Lactobacillus acidophilus*

Source	Sum of squares	*df*	Mean square	*F*‐value	*p*‐value
(a)					
Pb concentration	166.36	1	166.36	159.44	.066
*L. acidophilus* concentration	200.02	1	200.02	191.82	.0546
Contact time	249.3	1	249.3	226.61	.0631
Inoculation temperature	928.12	1	928.12	882.22	.1366
Shaking rate	0.1045	1	0.1045	2.88	.412
(b)					
Cd concentration	186.56	1	186.56	150.04	.059
*L. acidophilus* concentration	240.12	1	240.12	188.02	.0606
Contact time	255.3	1	255.3	230.11	.0731
Inoculation temperature	983.32	1	983.32	855.05	.1366
Shaking rate	0.1142	1	0.1142	3.03	.4014

### 3.2 Main affecting factors in Pb and Cd bioremoval by *Lactobacillus acidophilus*

#### 3.2.1 Effect of contact time and *Lactobacillus acidophilus* concentration

**FIGURE 1:**
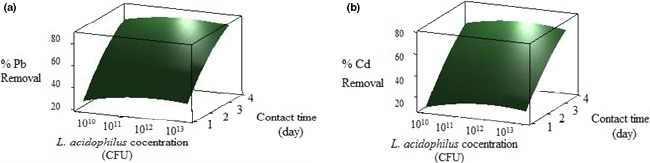
Interactive effects of contact time and *Lactobacillus acidophilus* concentration dosage on Pb (a) and Cd (b) removal

In this study, the ability of *L. acidophilus* concentrations (range of 10^10^–10^13^ CFU) on Pb and Cd bioremoval efficiency during the contact times (1–4 days) was evaluated. Figure 1a shows the Pb and Figure 1b Cd absorption by *L. acidophilus* at different contact times. As Figure 1a and b shows, the maximum binding rate of Pb and Cd occurred in the 4th day, respectively. The removal efficiency of these heavy metals first increased by rising the bacterial biomass and contact time and reached to the maximum level, then by the further increase of bacterial biomass, a light decrease in removal levels occurred.

In general, heavy metal biosorption mechanism is complicated and metal binding happens through the ion exchange in cell walls’ peptidoglycan and teichoic acid, the precipitation or the ligand formation (Zoghi et al., 2014). The cell walls of *L. acidophilus* contain a thick layer of teichoic acid, peptidoglycan, and exopolysaccharides. The functional groups such as hydroxyl, carboxyl, and phosphate create the negative charges. So, the bacteria would be able to absorb the heavy metals’ cations (Halttunen et al., 2007; Wang et al., 2015; Wang, Wang, Cheng, Bian, & Guo, 2017). It has been reported that metal binding occurs on the bacteria cell surface with no energy consumption (Halttunen et al., 2008).

In this study, the highest Pb and Cd removal efficiency was 80% and 72%, respectively, in biomass of 1 × 10^12^ CFU in the 3rd day. The difference in sorption of these two metals could be described as their ionic radius is different (ionic radius of Cd < Pb) (Bekri‐Abbes, Bayoudh, & Baklou, 2006). Similar studies reported that LAB showed the same results for increasing biosorption by passing the time (Halttunen et al., 2007; Rayes, 2012).

As shown in Figure 1 a and b, Pb and Cd bioremoval increased by enhancing the contact time from 1st to 4th day as well as rising the bacteria concentration. The optimum level of *L. acidophilus* was 1 × 10^12^ CFU.

#### 3.2.2 Effect of metal concentration and *Lactobacillus acidophilus* concentration

**FIGURE 2:**
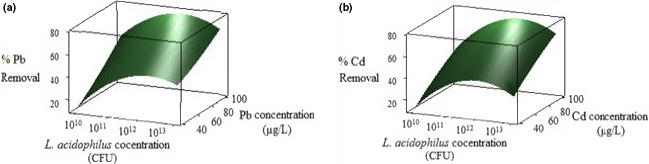
Interactive effect of metal concentrationand *Lactobacillus acidophilus* concentration and on Pb (a) and Cd (b) removal

The effect of initial metal concentration and *L. acidophilus* concentration on the bioremoval in the range of 10^10^–10^13^ CFU and 40–100 µg/L was investigated (Figure 2a and b). The results represented that by rising the metal concentration, the absorption increased. As shown in Figure 2a and b, by increasing *L. acidophilus* concentration up to 1 × 10^12^ CFU, the removal efficiency enhanced. The maximum Pb and Cd removal efficiency, 75% and 72%, respectively, was observed at the concentration of 100 µg/L and the biomass concentration of 1 × 10^12^ CFU.

*Lactobacillus acidophilus* has been reported to have a high affiliation for biosorption of heavy metals (Bhakta et al., 2012; Kinoshita et al., 2013). Heavy metals’ bioremoval is a surface process due to the binding of metal cations to the anionic functional groups and it also depends on the capacity of the bacteria strains and the metal electronegativity (Kang, Kwon, & So, 2016). LAB have some polymers like lipoteichoic acid that can be responsible for the interactions between the heavy metals and the negative charge of bacteria surface (Alcántara et al., 2017; Zoghi et al., 2014).

In this study, it is observed that by rising the metal concentration, the absorption to the bacteria receptors would also increase, which results in the higher biosorption levels (Dobrowolski, Szcześ, Czemierska, & Jarosz‐Wikołazka, 2017; Halttunen et al., 2007).

According to our findings, Pb and Cd biosorption efficiency enhanced by increasing the metal concentration (from 40 to 100 µg/L). The important factors in bioremoval as shown in Figure 2a and b are the *L. acidophilus* concentration as well as Pb and Cd concentrations and their optimum levels are 100 µg/L and 1 × 10^12^ CFU for the maximum level of the biosorption (75%). There are some studies in accordance with our results, Dobrowolski et al. (2017), Allam, Ali, Samya, and Abd‐Elrahman (2015), Akhmetsadykova et al. (2013), Bhakta et al. (2012) and Halttunen et al. (2008), as the absorption would increase by enhancing the bacterial biomass. Also by increasing the metal concentration, the bioremoval would enhance as observed in some studies by Pugazhendhi, Boovaragamoorthy, Ranganathan, Naushad, and Kaliannan (2018), Massoud et al. (2020), Shameer (2016), Kinoshita et al. (2013), and Halttunen et al. (2008).

### 3.3 Isotherm studies

The ability of *L. acidophilus* (10^12^ CFU/ml) for Pb and Cd bioremoval was determined at different metals initial concentrations (20, 40, 60, 80, and 100 µg/L). The biosorption isotherms are evaluated by the isotherm models of Langmuir and Freundlich. The regression coefficient (*R* ^2^) shows the best isotherm explaining Pb and Cd biosorption by *L. acidophilus*. All the tests were done in three replications.

One of the most common models that used in scientific studies is Langmuir model. The Langmuir equation is correct for monolayer absorption by the following equation (Langmuir, 1918):

(2) $$C_{e}/Q_{e}=1/\left(K*Q_{\max}\right)+C_{e}/Q_{\max}$$

where C_e_ (µg/L) is the equilibrium metal concentration in milk, Q_e_ (µg/L) is the amount of metal in absorbing process, Q_max_ (µg/L) is the maximum metal absorption in high C_e_ level, and K_L_ (L/µg) is the Langmuir constant. The C_e_/Q_e_ versus C_e_ indicates a straight line of slope 1/Q_max_ and also intercept of 1/K_L_Q_max_.

The Freundlich equation is using the following equation (Freundlich, 1906):

(3) $${\text{Ln Q}}_{e}={\text{Ln K}}_{f}+1/{\text{n Ln C}}_{e}$$

where the parameters n and K_f_ are the Freundlich constants. The n and K_f_ are defined from the linear plot of ln Q_e_ versus ln C_e_. Freundlich equation varies with the materials heterogeneity.

**FIGURE 3:**
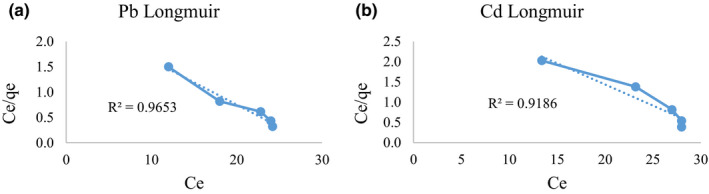
Langmuir absorption isotherm curve for Pb (a) and Cd (b)

**FIGURE 4:**
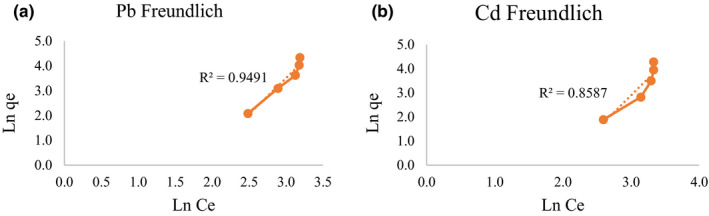
Freundlich absorption isotherm curve for Pb (a) and Cd (b)

As shown in Figures 3a,b and 4a,b, the biosorption increased by increasing the initial Pb and Cd concentrations. By having more metal concentration, more possible contact situations for metal ions and bacteria's functional groups would be presented (Jadán‐Piedra et al., 2017). By comparing the *R* ^2^ values in Langmuir and Freundlich models, it was observed that for both Pb and Cd, Langmuir isotherm model represented better fit than Freundlich model. It is confirmed that Langmuir equation is correct for monolayer absorption on surface binding. The higher correlation coefficient in Langmuir model indicates that both Pb and Cd absorptions obey the Langmuir isotherm model so these metal absorption process by *L. acidophilus* is a monolayer type of absorption on the surface of this bacterium.

## 4 CONCLUSION

Nowadays, heavy metals’ presence in food and water is a serious health concern all around the world. Pb and Cd are two toxic heavy metals for people. Milk is the most used dairy products that should be safe for consumption. In this project, Pb and Cd were removed from *L. acidophilus* successfully. The biosorption increased by increasing the bacteria and metal concentrations as well as the contact time. This study revealed that *L. acidophilus* is a natural effective biosorbent for Pb and Cd removal in very low concentration levels (ppb) from milk. Our findings are as the first step of investigating the ability of heavy metals binding by LABs in milk. There is still a need for more studies around other LAB strains in milk and other foods to eliminate the harmful effects of the toxic heavy metals.

## CONFLICT OF INTEREST

The authors declare that there is no conflict of interest.
